# *Notes from the Field:* Verona Integron-Encoded Metallo-β-Lactamase–Producing Carbapenem-Resistant *Pseudomonas aeruginosa* Infections in U.S. Residents Associated with Invasive Medical Procedures in Mexico, 2015–2018

**DOI:** 10.15585/mmwr.mm6820a4

**Published:** 2019-05-24

**Authors:** Ian Kracalik, Cal Ham, Amanda R. Smith, Maureen Vowles, Kelly Kauber, Melba Zambrano, Gretchen Rodriguez, Kelley Garner, Kaitlyn Chorbi, P. Maureen Cassidy, Shannon McBee, Rhett Stoney, Allison C. Brown, Kathleen Moser, Margarita E. Villarino, Maroya Spalding Walters

**Affiliations:** ^1^Division of Healthcare Quality Promotion, National Center for Emerging and Zoonotic Infectious Diseases, CDC; ^2^Epidemic Intelligence Service, CDC; ^3^Utah Department of Health; ^4^Washington State Department of Health; ^5^Texas Department of State Health Services; ^6^Arkansas Department of Health; ^7^Arizona Department of Health Services; ^8^Public Health Division, Oregon Health Authority; ^9^West Virginia Department of Health and Human Resources; ^10^Division of Global Migration and Quarantine, National Center for Emerging and Zoonotic Infectious Diseases, CDC.

Verona integron-encoded metallo-β-lactamase–producing carbapenem-resistant *Pseudomonas aeruginosa* (VIM-CRPA) and other carbapenemase-producing organisms represent an emerging U.S. public health threat because of high levels of antibiotic resistance and the potential for rapid spread in health care facilities (*1*,*2*). During September 18–November 19, 2018, CDC received 31 reports of VIM-CRPA through the Antibiotic Resistance Laboratory Network. Six cases (19%) occurred in U.S. patients who had recently undergone invasive medical procedures in Mexico. To identify additional cases (defined as isolation of VIM-CRPA from a patient who had an invasive procedure in Mexico in the month preceding specimen collection), CDC and state partners posted an Epi-X alert on November 19, 2018, and issued notifications through the Emerging Infections Network and to medical professional societies. As of January 18, 2019, a total of 12 cases had been identified in seven states, including four in Utah, three in Washington, and one each in Arizona, Arkansas, Oregon, Texas, and West Virginia; specimen collection months ranged from November 2015 through December 2018 (Figure).

**FIGURE:**
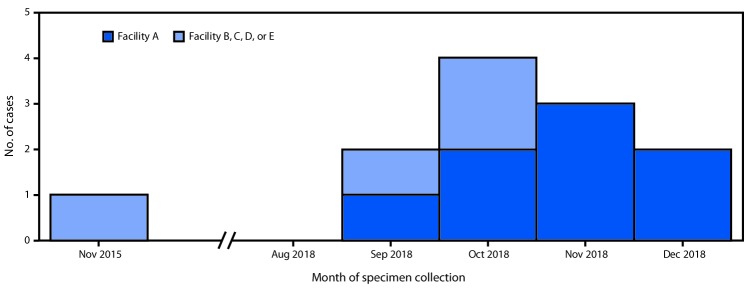
Number of U.S. patients (N = 12) who developed Verona integron-encoded metallo-β-lactamase–producing carbapenem-resistant *Pseudomonas aeruginosa* infections after surgical procedures in Tijuana, Mexico, by surgical facility and month of specimen collection — November 2015–December 2018

Among the 11 patients whose age was reported, the median age was 39 years (range = 29–62 years); among seven patients whose sex was reported, six were women. Eleven of the 12 patients were medical tourists (i.e., persons whose primary purpose for international travel was medical care) who underwent bariatric surgery. One patient, who was not a medical tourist, underwent endoscopic retrograde cholangiopancreatography in September 2018, after becoming ill while traveling.

Patients reported procedures at five hospitals in Tijuana, Baja California, Mexico, including facility A, where eight patients had bariatric surgery; the four other facilities (B–E) were identified by one patient each. The median interval from procedure to specimen collection was 11 days (range = 7–22 days); culture sources reported for 10 patients included the incision site (eight), blood (one), and an intra-abdominal abscess (one). Six patients were hospitalized in the United States for their VIM-CRPA infection. The patient who had a bloodstream infection died. Following notification by CDC, the Secretariat of Health of Mexico performed an onsite infection control assessment at facility A in December 2018 that identified numerous infection control breaches. A travel notice was posted on the CDC website from January to May 2019 informing U.S. residents of the risks associated with invasive procedures at facility A and recommending against surgery at facility A until the outbreak was over.*

This investigation highlights the potential for acquiring infections with highly antibiotic-resistant organisms not commonly found in the United States when receiving health care abroad that, once imported into this country, can spread within U.S. health care facilities. Persons considering medical care abroad should 1) visit a travel medicine specialist for advice tailored to their specific health needs at least 1 month before departure (e.g., current medical conditions should be well-controlled, travelers should have enough medication for the duration of their trip, and all medical tourists should be up-to-date on all routine vaccinations and consider immunization against hepatitis B virus); 2) check the qualifications of the providers who will be doing the procedure and the credentials of the facility, remembering that foreign standards for health care providers and facilities might be different from those of the United States; and 3) be cognizant that all medical and surgical procedures carry attendant risks (*3*).^†^^,^^ §^

Patients who become ill after returning to the United States following medical treatment abroad should report any hospitalizations or other medical care to their medical providers. Providers caring for patients who have undergone medical procedures abroad should obtain cultures when clinically appropriate, perform antimicrobial susceptibility testing to guide treatment, and test for carbapenemases in indicated carbapenem-resistant gram-negative bacteria (i.e., *Acinetobacter* spp., *Pseudomonas* spp., and Enterobacteriaceae). Any patient with an overnight stay in a health care facility outside the United States in the preceding 6 months should undergo rectal screening for carbapenemases on admission to a U.S. health care facility. Carbapenemase testing for carbapenem-resistant Enterobacteriaceae (CRE) and CRPA and rectal screening for carbapenemases are available free of charge via the Antibiotic Resistance Laboratory Network.^¶^

This investigation also underscores the importance of testing for the presence of carbapenemases in carbapenem-resistant *P. aeruginosa*. In the United States, carbapenemases are less frequently the cause of carbapenem-resistance in *P. aeruginosa* than they are in carbapenem-resistant Enterobacteriaceae (*4*). Because bacteria like *P. aeruginosa* can potentially harbor carbapenemase-producing genes, which are able to transfer antibiotic resistance to other organisms, early detection of carbapenemase-producing CRPA and associated public health responses might prevent spread of these resistant organisms. Clinical laboratories with capacity for carbapenemase testing should consider testing for both CRPA and CRE. For any patients infected or colonized with carbapenemase-producing organisms, CDC recommends implementation of infection control precautions to limit potential spread.**

## Footnotes

*
https://wwwnc.cdc.gov/travel/page/medical-tourism .

†
https://www.cdc.gov/features/medicaltourism/index.html .

§ https://stacks.cdc.gov/view/cdc/25250.

¶ https://www.cdc.gov/drugresistance/solutions-initiative/ar-lab-network.html.

** https://www.cdc.gov/hai/organisms/cre/cre-toolkit/index.html.
